# A qualitative exploration of travel-related risk behaviours of injection drug users from two Slovene regions

**DOI:** 10.1186/1477-7517-8-8

**Published:** 2011-04-17

**Authors:** Tatja Kostnapfel, Igor Švab, Danica P Rotar

**Affiliations:** 1 Public Health Institute of Ljubljana, Slovenia; 2 University of Ljubljana, Faculty of Medicine, Department of Family Medicine, Ljubljana, Slovenia

**Keywords:** travel-related risk behaviours, injection drug users, qualitative study, semi-structured interview

## Abstract

This qualitative study of travel-related risk behaviours of Slovene injection drug users was based on interviews with individuals enrolled in drug addiction treatment programmes run by three regional centres for prevention and treatment of drug addiction. The primary objective of the study was to analyse behaviour patterns and practices of injection drug users during travel.

**Methods:**

Travel-related problems of Slovene injection drug users were identified on the basis of data obtained by 25 in-depth interviews. A semi-structured questionnaire with 13 open-ended questions was developed after a preliminary study and review of the literature, and on the basis of experience with the treatment of drug addiction in Slovenia.

**Results:**

The sample comprised 25 individuals, 18 men and seven women, aged 25 to 53 years. The interviews were 10 to 30 minutes long. The results obtained were presented as identified risk behaviours. Five categories were generated, providing information on the following topics: procurement of illicit drugs, criminal acts/environment, HIV and hepatitis B and C infections, storage and transport of substitution medication and pre-travel health protection. The first three categories comprise the injection drug users' risk behaviours that are most frequently explored in the literature. The other two categories - storage and transport of medication across the border and pre-travel health protection - reflect national specificities and the effectiveness of substitution treatment programmes. The majority of participants denied having shared needles and other injecting equipment when travelling. Participants who had no doctor's certificate had recourse to various forms of risk behaviour, finding a number of ways to hide the medication at the border.

**Conclusion:**

This qualitative study provides insight into potential travel-related risk behaviour of injection drug users from two Slovene regions - central and coastal. The potential value of this qualitative study is primarily in the identification of potential risk behaviour of Slovene injection drug users travelling abroad. The study shows that injection drug users' experiences can contribute to better and more efficient treatment of drug addiction in Slovenia.

## Background

Estimates of the prevalence of illicit drug use and related health risks in Slovenia and the formulation of harm reduction strategies should be based on accurate analysis of the current situtation at various levels [1,2]. An estimated 7,500 individuals use drugs in a problematic manner [3]. Risks associated with sharing needles and syringes, mixing drugs (speedball), vascular injuries and unprotected sex are most frequently reported by drug users in Slovenia [2,4].

According to the 2008 data provided by 18 Slovene centres for prevention and treatment of drug addiction (CPTDAs), 3,332 of a total of 4,429 individuals were enrolled in opoid substitution treatment programmes (OSTP)[2]. The first CPTDA was founded in Koper to address the issue of dramatically increasing illicit drug use at the Slovene coast at the beginning of 1990 [5-7]. During our study, treatment of drug addiction was provided for 583 patients in Ljubljana, 236 in Koper and 191 in Piran, i.e., for 1,010 patients or nearly one-third of the total number of individuals enrolled in OSTP [8]. In Slovenia, as in other countries, methadone is the most commonly used medication for treatment of opoid addiction. The treatment is directed towards decreasing illicit drug use and reducing risks associated with problematic drug use, especially the risk of overdose and infection with HIV and hepatitis B and C viruses [3,5,9].

Physicians have full authority to dispense substitution medication to their patients. They may prescribe and dispense several takeaway doses of methadone to patients planning to travel outside their home environment [10]. According to the treatment protocol currently used in Slovenia, intending travellers are allowed to take with them a 14-day supply of medication. In addition, they are given a treatment certificate, required for transfer of substitution medication across the border [5,6].

Injecting drug users (IDUs) who have decided to enter an OSTP have to meet the programme requirements. Given that ordinary life situations may pose risks to these individuals, travel is another dimension of this issue in that it can lead to increased risk of exposure to risky behaviours [11,12]. IDUs face many problems when travelling; these may be due either to their behaviour and habits or to legal institutional, procedural and broader social factors, characteristic of destination countries [13].

IDUs most commonly decide to travel in order to escape legal problems or social pressures in their home environment, to enter a drug treatment programme abroad, or to conduct illegal activities related to the procurement and selling of drugs [12]. Exposure to HIV and to hepatitis B and C virus infections is the most frequent risk described in earlier research. Individuals that are most at risk are heroin users, who share injecting equipment, engage in unprotected sex or have multiple sex partners [1,12,14]. Another risk factor is the unknown environment in destination countries. Most of these individuals find themselves in high-risk situations because they lack money to purchase drugs and, when faced with an abstinence crisis, they are very likely to engage in criminal activities [15-17].

Risk-taking behavioural patterns of IDUs depend on both the individual and on the surrounding social environment [18,19]. Their decision to engage in risk-taking behaviour is thus not made only on the basis of critical reflection, it is more often determined by social factors [20]. Individual patterns of illicit drug users' risk-taking behaviour have been explored in several studies [12,20-22]. Many of these studies are ethnographic in nature and use a qualitative approach to data collection and processing, with the aim of improving understanding of illicit drug use and of providing answers to this problem [23-26].

An individual's activities constitute the first group of travel-related risks. IDUs are most likely to be exposed to the risk of HIV and/or hepatitis B and C infection [4,14,27,28] when travelling, primarily because of sharing injecting equipment and engaging in unprotected sex.

The HIV infection rate in Slovenia is low, i.e., less than one person per 1,000 of the population. The rate has shown a steady upward trend, however: 232 cases of HIV infection and 28 AIDS-related deaths were documented during the period 2000-2009 [29]. A study conducted in 2008 among voluntarily tested IDUs who had access to needle exchange programmes identified HIV infection in less than 1% of participants; 4.2% had hepatitis B infection and 22.3% were infected with the hepatitis C virus [30].

Lee and co-authors explored the travel experiences of a sample of 160 drug users and 44 non-drug users recruited as part of a study of HIV infection risks. Of the sample, 47% (96/204) reported travel experiences in the previous ten years. Drug injecting, safety of sex, number of sexual partners and duration of travel were investigated in association with drug use and HIV serostatus. Two significant relationships emerged: travelling drug users were more likely to inject drugs and to set off on longer trips than non-drug users. No statistically singificant differences in sex risk behaviour during travel were found between drug users and non-drug users or between drug-injectors and non-injectors. A comparison between risk behaviours undertaken at home and when travelling revealed significant differences in drug injection risks [12].

The environment is another risk factor for IDUs. Increased risk is associated with a number of factors, including poverty, joblessness, poor housing conditions, educational disadvantage, overpopulation and criminality [18].

Risks in the third category are related to the country from which a traveller comes. IDUs in Slovenia who are frequent travellers are offered information in CPTDAs on how to prepare for travel [3].

Since the prohibitory model for drug treatment, which stresses total abstinence as the final treatment target, has been losing credibility, alternative forms of counselling will have to be considered in the context of harm-reduction policy [1,5,10]. The aims include: providing better information about potential risks of disease and specific features of destination countries, about the required medical certificate and the risks associated with the transport of substitution medications across borders [7,14].

The objective of this study was to explore the behaviour patterns and travel experiences of IDUs during travel and improved harm-reduction strategies for drug users when travelling. The findings being aimed at stimulating further research into the control of travel-related risks.

## Methods

We present a qualitative study of 25 in-depth interviews conducted with IDUs involved in OSTP in Ljubljana, Koper and Piran CPTDAs [31]. The personnel of these CPTDAs were asked to help us make contact with this hard-to-reach population group. Interviews were conducted on a voluntary basis. The study inclusion criterion was travel abroad during drug being treated in OSTP in year 2009.

Data were collected using in-depth semi-structured interviews, including 13 open-ended questions. A semi-structured questionnaire was developed after a preliminary study and review of the literature and on the basis of experience with the treatment of opoid addiction in Slovenia [5-7].

Study participants were given written information about the study and were asked to allow digital recording and note taking. Discussions, participants' names, comments and answers remained confidential. All participants were able to answer all questions.

The topics covered in interviews included reasons for drug use and seeking medical counselling, description of drug injection equipment and behaviours, problems arising during travel and during transfer of substitution medication across borders, travel-related risk behaviour and type of assistance available abroad.

The interviews were conducted in Slovene. Verbatim transcriptions of quotes extracted from interviews were done by native Slovene speakers. Data were digitally recorded and transcribed [31,32].

### Data collection/analysis

Qualitative data collected between May and July 2009 were used.

Interview transcripts were read and processed by two independent investigators. Researchers used manual coding of basic textual material.

We analysed interview transcripts and searched for pre-determined words and phrases that best matched the answers to 13 questions. The search for pre-determined answers to each question was conducted over the entire text of the interview, the frequency depending on the number of topics searched for. We identified 57 codes likely to describe common characteristics of drug users [27,33].

The selection of quotations and their codes was done together with a comparison of respondents. Individual categories of responses were thus clarified in terms of importance, similarities and differences.

Codes were generated regarding travel-related problems reported by the Slovene IDUs interviewed. Categories are the extraction of behaviour pattern codes.

The qualitative database (interview transcripts) was broken down, and data were shown separately for each participant. Next, larger topics that connected similar answers were formed.

The coding scheme thus consisted of three steps, using the principle of progression from general (large) to ever-narrower subtopics. The coded contents were then entered into a theoretically devised risk factor frame.

### Ethical considerations

The study was conducted according to the guidelines of the Medical Ethics Committee of the Republic of Slovenia and was approved by this body in August 2008. Study participants gave informed consent to audiotaping and a literal transcription of interviews.

## Results

Interviews lasted 10 to 30 minutes, 387 minutes in total. The sample included 25 participants, 18 males and 7 females, ranging in age from 28 to 53 years. Some participants made trips to distant locations, mostly in Asia and America, but only stayed there for a month or less, whereas others travelled to Europe and/or other continents and stayed there, for various reasons, for several months. Some participants set off on a trip with no fixed plans concerning the destination and length of travel; in these cases, drugs were the principal motivation behind travel:

Categories of risk-taking behavious are: procurement of illicit drugs, criminal acts/environment, HIV and hepatitis B and C virus infections, storage and transport of substitution medication and pre-travel health protection.

### Procurement of illicit drugs

Procurement of illicit drugs constitutes the first category of risk-taking behaviour, reported by six study participants. For three of them, procuring drugs was the only motivation behind travel.

Generally, they had no difficulty procuring drugs, although this activity invariably put them into various high-risk situations, which reportedly happened in both European and distant Asian destination countries alike.

*"Drugs were the motivation behind all my travelling - Pakistan, Bangladesh, India, Thailand, most often. (...) There was no problem whatsoever to get it there; at that time, every carriage driver and, where there were tourists, every taxi driver had a pack and he waved to you if you were interested. For example, in Pakistan, India, especially Goa, you had no problem whatsoever... fifteen approached you before you managed to go up to any of them.(...)"(male, age 48)*.

*" Now, you seek and you find. Even when I went for the first time, it didn't take long. I think it was more difficult, 'cause it's not like in Europe, like it used to be in Holland, they don't sell in the street...they didn't at that time...but...who seeks, I think, always finds (...) I was attacked in Basel, in Rome, in Vienna"(male, age 50)*.

### Criminal acts/environment

Criminal acts belong to the second category of risk behaviour. None of the study participants reported committing a criminal offence to get money for drugs. Those who did engage in criminal activity said they acted spontaneously. Illegal activities were sometimes the goal of their travels and also a means of earning some extra money. Two respondents stressed problems with the police and the criminal environment in which they found themselves when procuring and/or selling drugs.

*"For instance, they know me so well in Dimitrograd that the Serbian custom officers asked me jokingly where I had my 200 grams for my own use. They told me they knew I didn't come all the way from Ljubljana just to buy three pairs of jeans every three months. (...). So that... And then, when I was selling...., I had to avoid this, too....They knew me in Rome after a couple of months, and they often searched me"(male, age 50)*.

*" Yeah, smuggling is most risky. And it used to end badly, too. ...well, in Germany, an Italian guy gave me away...it was about being betrayed most of the time. And in Germany, I once shut myself in a cellar, the cops found it but seeing my injection punctures, they thought. (...). I used to cross the border of Myanmar; I went illegally across two hills or so - once I nearly got killed - to buy for half the price, when I was short of money"(male, age 48)*.

Some other activities not directly related to drugs were also identified as criminal. Two study participants engaged in the illegal transport of people across a border and one used forged bank cards to draw money. Drugs invariably emerged as an additional factor increasing the risk and the likelihood of unexpected events. The respondents were of the same opinion:

*"I've been to Croatia, Dalmata. I spent six months in prison in Italy. I went to Germany (...) I was taking people across. (...). Sometimes I had my own stuff, but I like didn't dare to carry it across the border. Though we went there through a hole, that Schengen border, and back across the border, I nevertheless, they searched me once, but they found nothing, luckily I'd stuck it inside my socks, it's only there they didn't look. They usually do, so I said then that I was lucky, but now, never more"(male, age 32)*.

### HIV and hepatitis B and C virus infections

The possibility of infection with HIV and hepatitis B and C viruses constitutes the third risk category. Some participants admitted to sharing drug injecting equipment with other drug users without thought because they had no sterile syringes and needles, thereby increasing their risk of getting infected. However, the majority denied sharing injecting equipment while travelling and reported that they did not run the risk of HIV and/or hepatitis B and C infection. Only two participants shared their injecting equipment while travelling, explaining that an abstinence crisis and non-availability of sterile needles and syringes were the main reasons for their taking risks.

*"... a used syringe - definitely don't know, if there is one who would, I mean, wash this syringe, hot water, don't know what, if there is bleach. (...). Yeah, I used it"(male, age 48)*.

*"Somebody else's? Yes, I did if I had none. We, once, we were five of us, we had one (needle), we were on one....Because there was no place and, you don't care, you can't, can you. Otherwise I exchanged, right, also had my own, but if there was no other option, me too"(male, age 50)*.

Two respondents, who travelled abroad alone and for an extended period of time, did not use condoms, simply because they did not have any when necessary.

One participant infected with hepatitis C was aware of his risk behaviour, but admitted to having often engaged in unprotected sex in the past. He also said that most injecting drug users in his home environment practiced unprotected sex.

*"Of course, unprotected sex, this has happened all the time, hasn't it, but now we're more aware, so I don't do it any longer. Even here in Metelkova, nobody will use protection but we're a little more aware now, nearly all of us have hepatitis C, some of us use it nevertheless"(male, age 36)*.

### Storage and transport of substitution medication

The fourth category comprised topics that respondents identified as key problems encountered in storing and transporting substitution medication across borders.

Eighteen of the 25 study participants reported having applied for and obtaining a medical certificate required for the transport of substitution medication prior to every border crossing. The reasons for not having the document were that customs never check the certificate and that occasionally they did not apply for a certificate because of negligence.

Participants who had no certificate had recourse to various forms of risk behaviour, finding a number of ways to hide the medication at the border.

The issue of drug storage emerged on several occasions. The majority of study participants were treated with liquid methadone, which is difficult to hide. They often put the drug mixed with fruit juice in a plastic bottle, but a problem arose when they started drinking and did not know how much liquid was left in the bottle, exactly what daily dose they had to take.

*"I never needed it (a certificate), it was not required really, but as realize now, it is required"(male, age 40)*.

*" And I received it (methadone) from a female doctor, she just trusted me, but therefore I had to smuggle it. So I put it into orange juice, right, threw one bottle and a sandwich into it and set off" (male, age 48)*.

*" So I preferred to hide it, I poured it in a bottle, a Fanta can once, and in a fruit juice bottle once, 'cause fruit juice is mixed with methadone and I mixed them together. I was afraid of problems, because Croatia, Italy, I don't know if they tolerate these things. I preferred to hide it"(male, age 31)*.

The respondents consider crossing the national border and undergoing customs control as high-risk situations. Some of them reported minor problems crossing the borders of some neighbouring countries. These are often also experienced by individuals with a valid cerificate for legal transport of substitution medication across the border.

*"Yes, as a matter of fact. I get, we get this certificate allowing us to carry a certain number of bottles across but, as they say, not all customs officers stick to it, I don't know in which countries, they refuse you entry, and it's said they had to pour it away, in Croatia, too"(male, age 28)*.

### Pre-travel health protection

The fifth category includes problems encountered by participants when preparing for travel. CPTDAs provide personal health protection, particularly vaccination against hepatitis, and offer information on healthcare services available abroad. Intending travellers may be referred to travel clinics operated within the network of healthcare centres. Travellers get information there on the destination country and potential health hazards, as well as on the health protection measures required for entry.

The main sources of information reported by participants included CPTDAs, some non-governmental organisations and advice from friends. The Internet was listed as a very important source of information about areas to which they were travelling. The major problem reported was lack of information and inadequate instructions on what travellers should do when they have run out of substitution medications.

*"I think I even called last year when we went to Thailand, that I called to ask about the pills in Thailand, but we got two different pieces of information. Some said it was necessary, and others said it was pointless, so we simply didn't..."(male, age 36)*.

*"Yes, but even here, in Ljubljana, no doctor will sign if you've run out of methadone, or if it has been stolen from you, you go to the emergency unit, but they already kick your ass at the door. You can't get methadone absolutely anywhere on Saturdays, if, let's say, somebody has stolen it from you. Nobody gives a damn, that's your problem. I wonder how these things would be abroad"(female, age 44)*.

## Discussion

This qualitative study provides insight into risk behaviours in which IDUs from two Slovene regions engaged when travelling abroad.

We identified five categories of travel-related risk behaviour. Drug procurement, criminal acts/environment and the risk of acquiring HIV and/or hepatitis B and C virus infection have been frequently explored in the literature as risk-taking behaviour patterns of IDUs [15,17,20,21]. Storage and transport of drugs across borders and pre-travel health protection include behaviour patterns that are related to national specificities and the implementation of national drug policy [1,3].

Despite numerous risks resulting from the interplay of individual and social factors, some participants consciously chose to set off on a trip, the only motivation behind their travel being to procure less expensive drugs. As a result, they were very likely to commit illegal activities and become involved in the criminal environment, in which drugs constitute both cause and effect of risk-taking behaviour [15,18]. Other criminal activities reported by study participants were related to their attempts to make fast and easy money, and involved transporting people across the border, drug dealing and credit card abuse. In all these situations, study participants were exposed to numerous threats of physical violence, clear evidence of risky nature of their behaviour.

The increased risk of infection with HIV and/or hepatitis B or C viruses in the study participants was attributable to their inconsiderate and irresponsible behaviour. Only two of them (male, age 48 and male, age 50), admitted to having taken risks during travel; their high-risk behaviour was confirmed by quotes from the interviews. The majority of participants denied having shared needles and other injecting equipment when travelling. Risk of infection is associated with unprotected sex.

The results showed, however, that the travel-related behaviour of the study participants was less risky and much more responsible and thoughtful than expected.

As reported by some investigators, 5% to 50% of short-term travellers engage in risk behaviour by having sex without using condoms; the percentage is higher for long-term travellers. HIV-infected individuals constitute an especially high-risk group [14,27,28]. Other authors maintain that 23.3% of persons travelling abroad have sex with new partners and (only) 58.1% of them use condoms consistently [28].

OSTP have been generally recognized as an efficient tool for reducing drug-related harm, criminal activity and individual health risk rates [5]. One of the characteristics of these programmes is that drug users who, for various reasons, cannot attend CPTDAs on a daily basis are granted takeaway doses of substitution medication for home use [10]. Providing of takeaway substitution medication in a form most suitable for travel has an important impact on its transport across borders.

Prescribing substitution medications for long-term trips in itself represents a risk if it contributes to substitution drug trading on the black market [3,10]. Most study participants reported travelling with a certificate required for the legal transport of drugs across a border. Two study participants (male, age 48 and male, age 50), who were prone to engaging in risk-taking behaviour and travelled long term, reported having problems with the transport of medication across borders; the inconveniences they experienced seemed to be attributable to individual risk factors [18].

The reliability of the results of this qualitative content analysis therefore depends mostly on the accuracy of collection procedures and on the way of conducting interviews and categorizing risk behaviour [34]. The issue of validity, which re-emerged in data interpretation and categorization of risk behaviour, was addressed by using the above described coding method and by including two independent investigators [32,33].

## Conclusion

The value of this qualitative research project is primarily in the identification of potential risk behaviours of Slovene IDUs travelling abroad, which included: sharing injecting equipment related to the non-availability of sterile needles and other injecting paraphernalia, unprotected sex, transport of substitute medication across the border, drug storage problems, drug procurement abroad and criminal acts.

In conclusion, Slovene IDUs do not take great risks while traveling, even when they talk about sex as a possible mode of transmission of various diseases. They have a good understanding of their illness (addiction) and try to adjust to all life situations to the greatest extent possible. They are often the target of various forms of discrimination and stigmatization but they mostly cope with the problem situations successfully, as evidenced by the fact that they have families and job, and that they travel. The study showed that Slovene IDUs behave reasonably while traveling and that they tend to avoid situations defined as risky in this report.

IDUs experiences can contribute to better and more efficient treatment of opioid addiction in Slovenia. Problems experienced by IDUs during international travel, and the identified risk behaviour patterns help us better to understand the specific needs of these individuals.

Interaction between service users and physicians and other CPTDAs staff seems particularly important, therefore further improvements would be welcome in this area. This opinion was also expressed by the study participants. The important role of supportive therapy, education of DUs, their relatives and partners, group therapy and psychosocial support should be mentioned in this context [35,36].

Study participants favour counselling offered by CPTDAs as part of pre-travel preparation. Further improvements were suggested in terms of (more) accurate information and a more flexible approach to the issue of takeaway substitution medication. In the participants' opinion, these improvements would reduce the risks that they had experienced while travelling.

## Abbreviations

CPTDA: Centre for the Prevention and Treatment of Drug Addiction; IDUs: injecting drug users; OSTP: opoid substitution treatment programmes

## Competing interests

The authors declare that they have no competing interests.

## Authors' contributions

TK made a substantial contribution to the conception and design of the study, and data collection and analysis, whereas IŠ and DRP were involved in drafting the manuscript and revising it critically and have given final approval of the version to be published.
